# Why Wasp Foundresses Change Nests: Relatedness, Dominance, and Nest Quality

**DOI:** 10.1371/journal.pone.0045386

**Published:** 2012-09-25

**Authors:** Perttu Seppä, David C. Queller, Joan E. Strassmann

**Affiliations:** Department of Biology, Washington University in St. Louis, St. Louis, Missouri, United States of America; University of Arizona, United States of America

## Abstract

The costs and benefits of different social options are best understood when individuals can be followed as they make different choices, something that can be difficult in social insects. In this detailed study, we follow overwintered females of the social wasp *Polistes carolina* through different nesting strategies in a stratified habitat where nest site quality varies with proximity to a foraging area, and genetic relatedness among females is known. Females may initiate nests, join nests temporarily or permanently, or abandon nests. Females can become helpers or egglayers, effectively workers or queens. What they actually do can be predicted by a combination of ecological and relatedness factors. Advantages through increased lifetime success of individuals and nests drives foundresses of the social wasp *Polistes* from solitary to social nest founding. We studied reproductive options of spring foundresses of *P. carolina* by monitoring individually-marked wasps and assessing reproductive success of each foundress by using DNA microsatellites. We examined what behavioral decisions foundresses make after relaxing a strong ecological constraint, shortage of nesting sites. We also look at the reproductive consequences of different behaviors. As in other *Polistes*, the most successful strategy for a foundress was to initiate a nest as early as possible and then accept others as subordinates. A common feature for many *P. carolina* foundresses was, however, that they reassessed their reproductive options by actively monitoring other nests at the field site and sometimes moving permanently to new nests should that offer better (inclusive) fitness prospects compared to their original nests. A clear motivation for moving to new nests was high genetic relatedness; by the end of the foundress period all females were on nests with full sisters.

## Introduction

Individuals have evolved to maximize their fitness, either by reproducing themselves, or by helping relatives, which carry their genes, to reproduce. [1], [2] In social groups, only a few individuals often monopolize actual reproduction. The others more or less voluntarily assume the role of helpers whose fitness is dependent on helping relatives, not on reproducing themselves. Two syndromes leading to this kind of advanced sociality have been recognized. “Fortress defenders” dominate a valuable resource, such as food, so staying home and not dispersing improves their possibilities for defending that resource. These include some social insect groups such as termites and social aphids, and also mole rats and social shrimp. On the other hand, ants, bees, and wasps, are “life insurers,” with a social life style that provides possibilities for extended parental care for non-independent young through overlapping generations. [3]–[5].

Reproductive division of labor has been taken to the extreme in social insects, with queens acting as a specialized reproductive caste in colonies with either totally or partly sterile workers. In most social insects, roles of queens and workers as well as colony structures are relatively fixed, which makes leaving the nest and moving to a new one an unrealistic option for the queens. In *Polistes* wasps, however, spring foundresses have a true opportunity to choose between solitary and social nesting, as well as the opportunity of revising their decisions should the original nesting choice prove to be inferior. This makes them ideal for studies of reproductive strategies.

Sometimes several females coexist and reproduce in social groups, which may be advantageous for the females if dispersal is too risky or if ecological constraints for single nesting are too strong. [6], [7] If so, we can ask what determines who gets to reproduce in the group. It may be good to help, but it is always better to be helped. This conflict is likely to be controlled, since joint nesting is very successful and common in social hymenoptera. Indeed, a common outcome is that reproductive rights are claimed based on direct competition between females, for instance in a dominance hierarchy [8], [9], or by a convention based on some asymmetry between the rivals, such as size, territory ownership or precedence. [10]–[12].

*Polistes carolina* is a cavity nester, so suitable nesting sites, such as hollow trees, are a scarce resource in the wild. Once found, a cavity can endure, so the wasps re-use them, even chewing down old nests in autumn in preparation for the new season. Consequently, *P. carolina* foundresses have evolved under circumstances of nest site shortage, which impacts both nesting decisions and the fitness consequences of those decisions. In an earlier paper, we established the existence of a strong constraint against solitary nest founding in *P. carolina*. We also showed that reproductive dominance appears to be determined by convention, that the foundress who actually initiates the nest usually also becomes the dominant reproducer, and that other foundresses join their natal nest mates at newly initiated nests. [10] The option of leaving a social group and nesting independently, as well as relatedness among co-breeders are important parameters in skew theories that model how reproduction should be partitioned. [13]–[15] We were able to measure these parameters, yet found no support for skew theory. [10].

Now we examine what nesting choices *P. carolina* spring foundresses make after relaxing a strong ecological constraint (nest-site shortage), and what consequences different choices may have for their fitness. Overwintered foundresses start new nests in the beginning of the season, either alone or in groups. In our study area, this takes place in March and foundresses take care of the brood through the pre-emergence period until first brood emerges, about two months later. The first brood consists almost exclusively of females [10], [16], who take over the worker tasks from foundresses. New sexual brood are produced only at the end of the season. [16].

**Figure 1:**
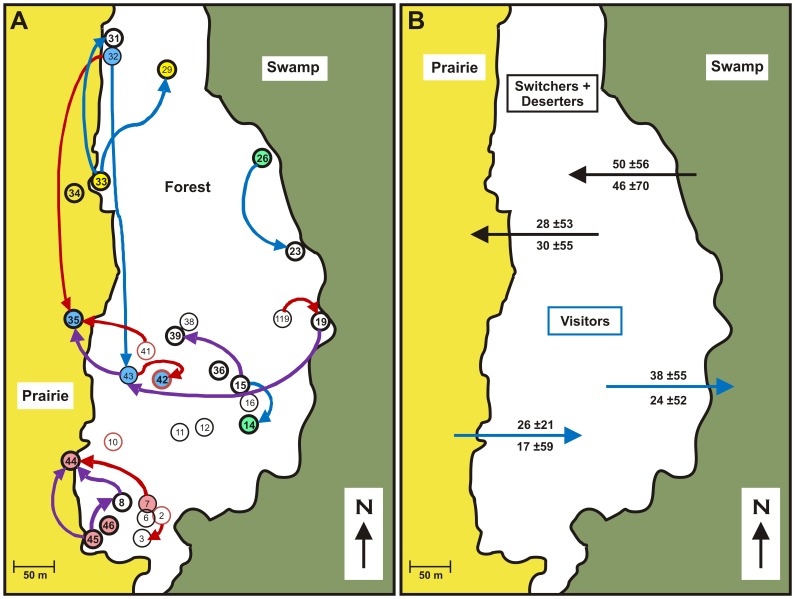
A map showing location of the study nests and moving of foundresses. The field site was a c. 10 ha section of pecan/oak forest in Brazos Bend State Park, TX. The yellow area on the left is an open prairie; the green area on the right is a swamp, superior and inferior foraging areas, respectively. In *panel A*, nests marked with a larger font and a bolded circle are nests that survived until the end of the field period. Nests with a smaller font and a non-bolded circle are the ones that failed before the end of the field period. A red circle shows the nests that were adopted during the field period. Movements of the wasps are indicated with arrows. Purple, red, and blue arrows show switching, deserting and visiting, respectively, with the arrowhead indicating the target nest. Nests inhabited by full sister foundresses are filled with consistent colors (green nests: 14, 26; yellow: 29, 33 34; red: 7, 44, 45, 46; blue: 32, 35, 42, 43); nests not filled with a color were inhabited by a single foundress or full sister foundresses restricted to that nest. In *panel B*, the arrows indicate the direction of the moves, black arrows show switching and deserting (combined) and blue arrows visiting. The figures above the arrows (mean ±SD) show how much movers increased/decreased their distance from/to prairie/swamp by moving between nests (in meters); the figures below the arrows are the expected increases in distance had the movers selected their target nests randomly; all increases in distance were as expected (Mann-Whitney, all P’s >0.31).

We had previously provided the wasps with nest boxes, which presumably attracted the vast majority of the population at our field site (see Figure 1). Consequently, our study provided a unique opportunity to monitor individually marked foundresses of almost an entire field population for the whole nest founding period. We monitored foundress behavior with direct observations and videocameras. We used DNA microsatellites to determine relatedness patterns in each nest and reproductive success of each foundress. This way we were able to assess each nesting choice and how they affected foundresses’ reproduction, obtaining a comprehensive picture of the dynamics of the foundress population during the nest founding stage. We show that many foundresses reassessed their reproductive options after nest initiation by moving to new nests where they joined full sisters, related by 0.75. Increasing the number of wasps on nests of high relatedness increased their survival and increased the numbers of reproductives they produced at the end of the season.

## Results

We detected 104 wasps in the nest boxes either through direct observations or by inferring their existence from the combined census and genetic data. We may have counted a very few foundresses more than once, if they were inferred by genetic data, also observed behaviorally, but not collected at the end so observations could not be tied to a genetic individual. We are primarily interested in foundress behavior, so a slight variance in final count will not change our results. Foundresses made a total of 125 nesting choices. Fifteen foundresses reassessed their reproductive options after making their initial decision, with nine and six foundresses making two and three choices, respectively. We divided the nesting choices to four major and three subcategories, which we list and define in Table 1.

**Table 1:** Definition and number of behavioral choices (n = 125) foundresses made during the pre-emergence period in the study population.

Behavior	Definition	#
1. Nest founding	Foundress was the first wasp observed on a new nest	30
2. Joining	Foundress seen or not seen on other nests shows up on an occupied nest	57
3. Adopting	Foundress seen or not seen on other nests shows up on an abandoned nest	4
4. Moving	Foundress seen on other nests moves to a new occupied nest	
4.1 Switching	Foundress moves permanently to a new nest, the original nest survives the move	6
4.2. Deserting	Foundress moves permanently to a new nest, the original nest fails	6
4.3. Visiting	Foundress moves to a nest where she is not a permanent resident, and later returns to the original nest	22

### Nest Founding and Joining

**Figure 2:**
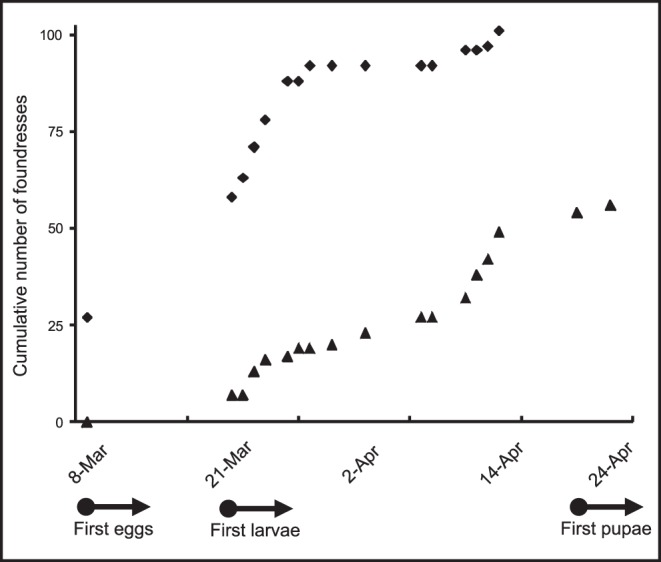
Cumulative number of foundresses appearing and disappearing from the population. Foundresses appearing (n = 104) are indicated as diamonds, foundresses disappearing from the population (n = 54) as triangles. Below the graph, the date when eggs, larvae and pupae started appearing in the nests is indicated.

A mated female first emerging from hibernation can initiate a new nest or join an established colony. Most foundresses appeared at the field site at the very beginning of the season. Ninety-two wasps (89%) had appeared by the end of March, and the remaining twelve wasps appeared in mid-April. Fifty-six (54%) foundresses disappeared from the population during the field period (Figure 2), probably due to natural death from predators such as robberflies, summer tanagers, and spiders. In independently founding wasps, such as *Polistes*, nest founding is an essential behavioral choice, because all subsequent nesting choices require that some foundresses successfully initiate nests. Twenty-four (80%) *P. carolina* nests were initiated by a single foundress. Six nests already had two to five foundresses at our first census. We could not distinguish nest founders from joiners in the latter nests, if indeed they were not initiated simultaneously. Twenty nests were initiated right at the beginning of the season before March 8 and the remaining ten were initiated by March 23. We call these early and late nests, respectively.

**Figure 3:**
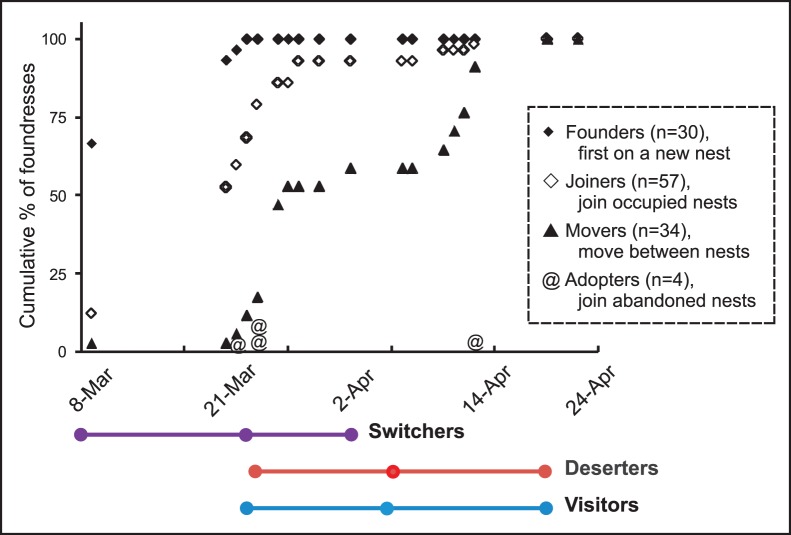
Cumulative frequency of foundress behaviors. Foundresses that initiate nests (n = 30) are indicated as filled diamonds, joining foundresses (n = 57) as open diamonds, and moving foundresses (n = 34) as filled triangles. In addition, adoptions are marked with @ signs on the day of adoption. Below the graph, the timing of moving events is divided into switching, deserting, and visiting (mean and range), showing that switchers moved earlier than other movers. A full explanation of the behaviors is given in Table 1.

Forty-seven foundresses *joined* 21 newly founded nests as their first choice, and ten as their second choice. The latter foundresses had previously founded (3), joined (5) or adopted (2) other nests, but abandoned them and moved to the target nest (see below). All but four joining events (93%) occurred during the first three weeks. All but one of the nests that eventually received joiners had received their first joiner by then. The last joining events occurred during week six (Figure 3).

An early start can be critical for nests as a whole to increase fitness, something indirectly supported by nest growth and survival patterns. Early nests were not more likely to survive than late nests (Early: 13 of 20, Late: 4 of 10, Fisher exact test *P* = 0.26). However, early nests attracted significantly more joiners (2.9±2.5 SD, n = 20 nests) than late nests (0.9±0.7 SD, n = 10; Mann-Whitney, *P* = 0.018). Nest founders that managed to attract joiners lived significantly longer (42.4±11.1 days, n = 22) than solitary foundresses (22.1±16.4 SD days, n = 8; Mann-Whitney, *P* = 0.002). Early nests that survived had an apparently larger number of cells at the end of the field period, although this difference was not significant (early: 17.3±4.3 (SD), n = 4; late: 26.2±13.4, n = 13; Mann-Whitney, *P* = 0.26). Nests with more joiners added more cells, an indicator of increased fitness (linear regression of the number of joiners vs. the final nest size: *b* = 0.72, *r* ^2^ = 0.52, *F* = 16.4, *P* = 0.001, n = 17).

Early foundresses also have the possibility to choose the best nest sites, which could lead to better survival and reproduction in those nests. The best nesting sites provided ready access to the prairie with its rich insect base as compared to the flooded swamp. Foraging wasps were frequently seen in the prairie, not in the swamp edges. Wasps leaving the nest flew in the direction of the prairie (unpublished observations). When prey was identifiable, it generally took the form of caterpillars from prairie forbs (unpublished observations). However, nests that survived (n = 17 nests) were not located at better sites,than the nests that failed (n = 13; distance to prairie: Mann-Whitney, *P = *0.74; distance to swamp: *P = *0.52). Yet, surviving nests grew larger the further away they were from the swamp, but this effect was also small and not quite significant in a stepwise regression model (*r* ^2^ = 0.20, *F* = 3.82, *P* = 0.069; distance to swamp: *b* = 0.01, *t* = 1.96, *P* = 0.069).

Based on the genetic data, augmented by census information, we grouped 71 spring foundresses into 19 full-sister groups. Most full-sister groups had just 1–2 foundresses inhabiting a single nest, but the two largest full-sister groups occupied four nests each (see Figure 1), making up more than one third of all foundresses residing at the field site. These two sisterhoods nested near the superior prairie foraging area (Mann-Whitney, *P* = 0.011) and further away form the swamp (Mann-Whitney, *P* = 0.029) than sisterhoods confined to just one or two nests (Figure 1).

### Adopting

Adopting a nest is a surprising behavior, because the brood will be unrelated to the adopters. It only makes sense because the first workers do not become reproductives, but instead work for the queen. Four foundresses adopted nests that had lost their previous residents within two days. This adoption rate was rather high, because only nine nests were available for adoption at any time after losing their original foundresses. Three nests were adopted during week three by foundresses that were new to the field site (Figure 3). These adoptions were not successful; they were either never joined (#2, #41) and the adopters switched to other nests, or an adopter managed to attract one joiner (#10) but the nest nevertheless failed two weeks later. During week six, all original residents of nest #42 disappeared. Two days later the nest was adopted by a foundress moving from a nearby nest, #43 (Figure 1). Another foundress joined her and they survived with their nest until the end of the field study. However, one of four adopters surviving is not statistically different from the survival of the whole wasp population (Fisher exact test, *P* = 0.41).

### Moving Foundresses

The initial nesting decision of females can be modified as conditions change. Previously we found that some foundresses moved between nests and showed that they mostly joined nests with related foundresses. [10] By combining behavioral observations with genetic information, we are now able to identify additional moving foundresses and are able to divide them into subclasses depending on the permanence of the move and the fate of the rejected nest (Table 1). Eleven foundresses made twelve moves (one foundress moved twice, Table S1, Figure 1). Five *switchers* moved six times between extant nests, and six *deserters* either caused the failure of the original nest or moved because the nest was destroyed (nest #32, Table S1, Figure 1). Twenty *visitors* made twenty-two visits to our study nests (two foundresses made two visits, Table S2, Figure 1). Visitors were either observed in the target nest only once (11 cases), or laid eggs in the target nest confirmed by genetically assigning progeny to foundresses who were not residents there (11 cases).

Foundresses moved and visited other nests frequently, but the different subclasses of females moved at different times (Figure 3), suggesting variable motives for moving. Switchers left their original nests first (average March 23), followed by visitors visiting other nests (April 5) and deserters leaving their nests (April 6, see Figure 3). Furthermore, the visits by visitors who did and did not reproduce in the target nest occurred at different times as well. The offspring of former visitors were already larvae at the time of collection (10/11 cases). Thus, these visits must have occurred no later than two weeks before the end of the field study. On the other hand, only 1/11 cases of the visits that did not lead to egg laying occurred before this cut-off, a highly significant difference (Fisher exact test, *P*<0.001).

**Figure 4:**
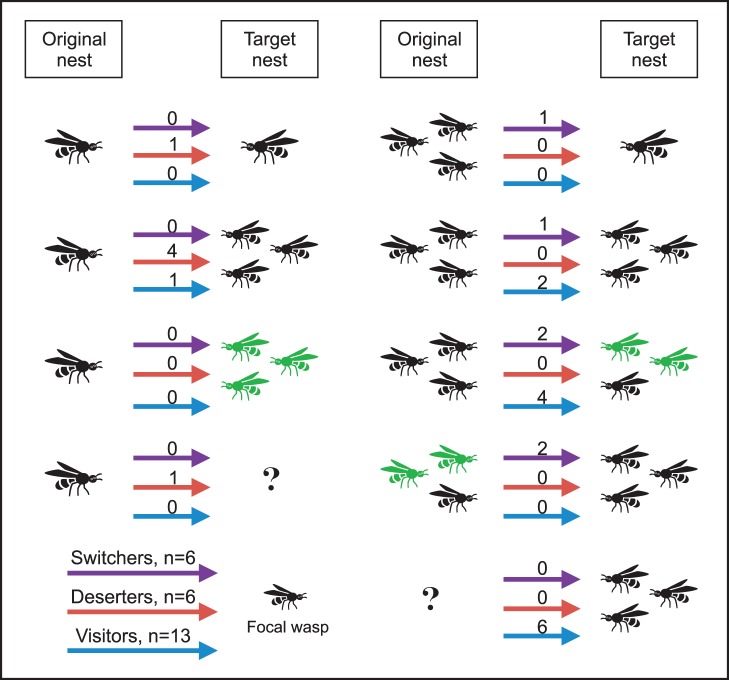
Relatedness of moving foundresses in the original and target nests. In the first column, the moving foundress was the only wasp at the original nest; in the second column the moving foundress was with other wasps at the original nest. Color of the wasps indicates relatedness of the moving wasp to other wasps in the original and target nests. The focal moving wasp is always marked as black and green wasps are ones not related to her but full sisters to each other; question marks indicate that relatedness among wasps could not be determined. Thus, a wasp moving to a target nest of black wasps only is joining full sisters. The figures indicate the observed number of moves for each category of movers. Both permanently moving foundresses (switchers + deserters) and visitors targeted their full sisters significantly more often than random (Table S1, S2, expected number of movers targeting full sisters: sw+des: 1/11; visitors: 1/13; Fisher exact test, *P* = 0.002 and *P* = 0.001, respectively).

**Figure 5:**
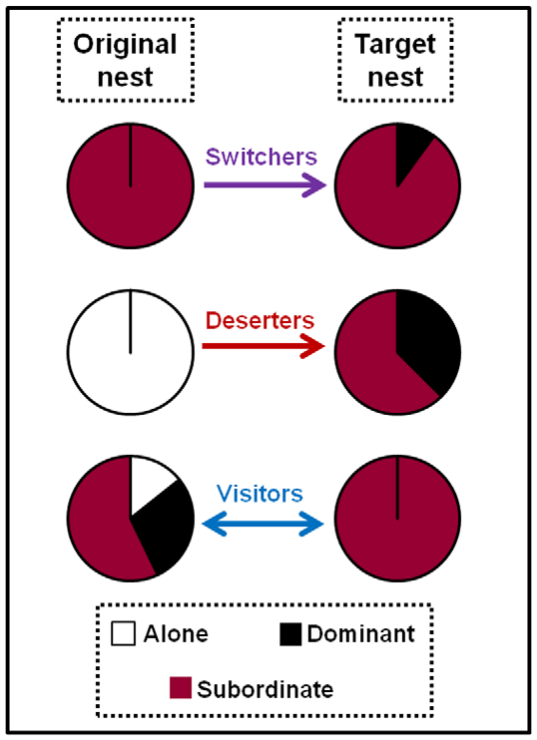
Proportion of movers that were alone, dominant, or subordinate foundresses at their original and target nests.

Moving foundresses joined as subordinates on nests controlled by their relatives and did not discriminate based on either nest size or location. Both original and target nests of the movers had an average number of cells and foundresses when compared to other nests on the day of each move, except that the target nest had a larger than average number of foundresses (Wilcoxon Signed Rank Test, *P* = 0.003, other *P*’s >0.48). Different classes of movers were similar in this respect (Kruskal-Wallis test: all *P*’s >0.66), except that switchers left significantly larger foundress associations (4–5) than deserters (always 1) or visitors (1–3) (Table S1, S2, Kruskal-Wallis test: χ^2^∶16.0, *P*<0.001; df: 2). Permanently moving foundresses targeted nests that were further away from the swamp while visiting foundresses targeted nests were further away from the prairie, but all moves were apparently directed as expected had the movers selected their target nests randomly (Figure 1). Finally, moving foundresses were usually either with their full sisters (switchers, visitors) or alone (deserters) at their original nests, but they joined and visited their full sisters more often than random (Figure 4, Tables S1, S2). Both switchers and deserters usually ended up as subordinates in their target nests and produced only a few of their own brood (Figure 5). The increase in brood production could be measured in switchers and was not significant (Mann Whitney, *P* = 0.37, n = 5, Table S1).

## Discussion

We succeeded in following detailed nesting decisions of paint-marked *P. carolina* foundresses by providing nest boxes, then monitoring the wasps that used them. We took censuses, made behavioral observations, including videotaping, and genotyped with DNA microsatellites to assess genetic relatedness and to detect surreptitious egg laying. For an individual foundress, the most successful reproductive strategy is to initiate a nest as early as possible, in the best available nesting site, and then accept other foundresses that join her nest since they generally only do so as subordinates. Joining consolidates females on nests, thereby increasing their growth and survival and, ultimately, sexual production at the end of the season. Because foundresses join nests dominated by their natal nest mates, related as full sisters, increased performance of the nest will benefit not only the direct fitness of the initial nest founder, but also the inclusive fitness of joining foundresses.

*Polistes* wasps are primitively eusocial without morphologically differentiated queen and worker castes. This means that, at least in some species, even workers have the option of leaving the nest, mating and nesting solitarily. This seldom happens in most species (but see [17]–[19]). In particular it is unlikely in *P. carolina,* since they are unique among Texas *Polistes* in not producing a few males in the first brood. [16] The lack of males means workers that might be selected to become egg layers would only be able to produce males from unfertilized eggs. Even spring foundresses often nest in associations, which suggests that social nesting provides strong benefits compared to solitary nesting, probably in the form of life insurance. [3], [4] Since solitary nests in *P. carolina* always fail [10], this species goes to extremes in this respect and its social nesting is not really facultative anymore but obligatory. So, in contrast to many other *Polistes*, sociality in *P. carolina* resembles in this respect more advanced social groups like honey bees, stingless bees, and army ants, where social life is obligatory at all stages.

### The Start of the Season: Nest Founding and Joining

An early start is decisive for *P. carolina* foundresses, because emerging early from hibernation allows them to dominate reproduction by starting nests, since queenship is determined by order of arrival. [10] Foundresses emerging early from hibernation can also secure the best nesting sites, the ones close to the main foraging area (prairie) and furthest away from the swamp. Supporting this, the largest sisterhoods inhabiting several nests were located on good-quality spots; nests further away from the swamp grew larger and wasps moved away from the swamp. Nest site fidelity would accentuate this relationship if more successful nests from the previous year were also farther from the swamp. Precise quality measures of nest sites, including microclimate, and exact measures of forage potential in different areas await further study.

Some of the initiated nests never got joined and so failed, which may be due to lack of suitable joiners. One of the strengths of our study was that we got to witness nesting choices that might not have been expressed had we not relaxed nest site constraints. We provided excess nest sites (nest boxes), something that is usually a scarce resource, so more foundresses probably attempted nest founding than might have done so had nesting sites been more constrained. This allowed us to see in more detail choice behaviors that might have otherwise taken the form of short contests, rather than beginning nests and leaving.

### Reassessment of Reproductive Options

A common feature for foundresses that failed to dominate egg laying in is that they actively monitored other nests, by visiting and sometimes also laying egg(s) in the nests they monitored. Most of the egg-laying in visited nests took place early in the season, when foundresses were busy building nests and probably spent relatively more time away from the nest compared to later in the season. Otherwise it is hard to imagine a visitor could sneak in an egg. Foundresses seemed to be generally well aware of other nests in the population; they knew where they were located and whether or not they were occupied by their natal nestmates, at least as far as we could tell by their visits. Many foundresses took advantage of this information, as a sizeable proportion of foundresses moved to new nests. We observed, for instance, that some foundresses first initiated nests by themselves, but later abandoned them and joined other nests after their own did not attract any joiners.

Because moving foundresses became non-egg laying subordinates at their new nests joining full sisters is crucially important. Since nests of non-relatives were in the same area, associating with natal nest mates is not just philopatric behavior but also an active choice. That females preferentially join natal nestmates on different new nests shows that foundresses recognize not only current nest mates but also natal nest mates. Some of the foundresses moved between related nests that were physically not particularly close to each other (Figure 1), suggesting that foundresses indeed knew where their natal nest mates were nesting.

In general, the benefits of staying at their original nest or joining a new one depend on both the likelihood that the nest will thrive (many wasps, close to prairie), and genetic relatedness of the joiner to the egg layer. The likely success of the target nest may surpass a female’s original nest especially when an additional joiner increases the workforce of the new nest. A subordinate may choose to help where helping makes the biggest difference. However, all movers, including the switchers, moved to random sized nests in terms of both nest size and the number of foundresses. However, two of five switchers moved to nests with higher per capita cell number as compared to their original nest (i.e. the target nests were relatively large for the number of foundresses taking care of them; data in Table S1). This suggests that motivation for switching to a new nest at least in those cases was to provide additional brood care where it was most needed. Since nests with only one foundress always failed, it made sense for those females to find relatives to join. [10].

Timing of leaving a nest and joining another is obviously crucial, particularly for deserters. If a foundress abandoned her original nest too early, she would trade away the possibility of staying with her original nest as a dominant foundress, should she be joined. But by leaving too late she would forfeit time she could spend raising related brood in a nest that would succeed. In our study population, deserters started abandoning their original nests only when the probability of getting joined diminished considerably, towards the end of week three. Only eight joining events (21%) happened after the first deserter abandoned her nest, and only four (7%) after the mean day of deserting. So, there is obviously a cut off when the probability of getting joined becomes so low that waiting for a joining foundress does not pay anymore, making abandoning the nest a more rewarding option. Switchers left their original nests considerably earlier, at a time that coincided well with the general activity at the nests during the first three weeks of the field period, when most foundresses also appeared and joined nests.

Some foundresses took advantage of other foundresses’ nest founding efforts and skipped the dangerous and energy consuming nest-founding stage by adopting newly-abandoned nests. It must be a lucrative option for a foundress that appears too late to initiate a successful nest, because partly raised brood would give an important head start for the adopter. [3] The problem that the adopted brood is unrelated to the adopter is not serious, because those brood would not become reproductive, but would instead rear the progeny of the new, unrelated queen. Furthermore, emerging in a nest dominated by an unrelated foundress most likely would not affect workers’ efficiency to perform their tasks, as the brood and unrelated foundresses would share the cues important for nestmate recognition (e.g. [20]). Almost half of the abandoned nests were actually adopted within a day or two, but only one adopted nest survived until the end of the field period. This was probably due to most adoptions also occurring too late to attract joiners, but does not rule out the possibility of recognition problems. In absolute terms, however, very few foundresses adopted abandoned nests, probably because *P. carolina* foundresses use cavities as nesting sites, and an opening of such a valuable resource must be quite rare. Thus, adopting may be an option that *P. carolina* foundresses rarely face in the wild.

### Breeder Group Composition in Social Wasps, and Beyond

Apart from *Polistes carolina*, detailed choices among incipient social groups have been characterized in only a few other species. Besides joining previously founded nests and disappearing due to natural causes, foundresses have been shown to switch nests in *Polistes dominula* [21] and *P. bellicosus*. [22] Foundresses can also take advantage of each other’s nest-founding efforts by usurping nests initiated and built by others, by taking them over (usurpation), as in *P. dominula* [21] and *P. biglumis* [23], [24], or by adopting recently abandoned nests, as in *P. dominula*. [18], [19], [21], [25] Breeder composition is also unstable in another group of primitively eusocial wasps, the *Stenogastrinae*. For instance, a considerable proportion of *Liostenogaster avolineata* females are “floaters”, who leave their natal nests and join or adopt other nests. [26], [27].

Breeder changes in other social insects are poorly known. In some ants, multiple unrelated queens initiate nests together (pleometrosis), but usually only one of them survives in a mature nest (e.g. [28], [29]). Colony lifespan is usually much longer than the lifespan of individuals in perennial social insect colonies with multiple queens. In many other *Polistinae* wasps (e.g. [30], [31]), bees [32] and ants [33], [34], females are typically recruited to their natal nests, with multiple egg-layers coexisting (secondary polygyny) but sometimes also succeeding each other (serial polygyny).

In mammals and birds, there is not a clear parallel to the incipient stages of social groups. The typical pattern is that young stay with their parents, with one sex staying more than the other. [35], [36] In the lion. for example, most daughters are recruited to their natal pride as new breeders eventually replacing the old breeders [36], [37], while breeding males are replaced by unrelated dispersing males. [37], [38] In cooperatively breeding birds, principal breeders in the group are replaced by unrelated dispersers rather non-dispersing offspring. [39], [40] Sometimes a helping male can also replace a principal male breeder (his father), but only if the female breeder (his mother) has been replaced first, as in the white-breasted thrasher (*Ramphocinclus brachyurus*). [41].

If we had simply visited our nests at the end of the foundress period, we would have found nests with multiple foundresses comprised exclusively of full sisters. We would have seen that full sister groups sometimes covered multiple nests, and that these tended to be near the best foraging areas. We would have missed a rare insight into all the decisions that led to this condition. We would have missed the females that tried independent nesting and gave it up. We would have missed the females on nests of non-relatives who subsequently moved. We would have missed many acts of movement and assessment which indicate how well the wasps know what their options are on neighboring nests. There is no substitute for careful, long term observations that combine behavioral, census, and genetic tools for understanding social decisions.

## Materials and Methods

Our study site was a 10-ha section of pecan/oak riparian forest, located at Brazos Bend State Park, near Houston, TX,. The field site was bordered by open prairie at one end and a swamp at the other (Figure 1). We had previously provided the wasps with about 50 wooden nest boxes. We believe that we were able to attract the vast majority of the population at our field site to the nest boxes, although we may have missed some foundresses that were nesting in natural cavities.

We found the first wasps in boxes without nests on February 23 1995 and the first nests appeared by March 8. We associated foundresses with their nests by individually marking 87 foundresses on 30 nests. We monitored the nests on average every two days for a fifty-day period (March 8 - April 28), which covered the nest founding stage from initiation until shortly before first workers emerged. During each census, we recorded the number and identity of foundresses present and the size of the nests (number of cells). If a given foundress was not observed on her nest on a given day, but was observed before and after that day, we assumed she was also present on the intervening day. Some of the nests failed during the study period. The failure was dated to the first day when no foundresses were seen on the nest, or to the first day the number of cells did not increase in the nest and there were no foundresses.

We also augmented the census data by videotaping 21 nests (mean 11 h, total 231 h) over two periods: early from 24 March to 15 April, and late from 24 to 26 April. We quantified the quality of the nesting sites (boxes) by measuring their distance from the swamp and the open open prairie bordering the field site (Figure 1). We regarded nesting close to the prairie as superior because wasps foraged for caterpillars in the fields.

At the end of the field period, we collected 46 foundresses and nests with brood (eggs and larvae) from seventeen successful nests. We genotyped all adults, all sperm samples dissected from their spermathecae, and 371 brood (90% of all brood) for seven DNA microsatellite loci [42], using standard genetic methods [43] (see also [10]). Genetic data were used to assign brood to their mothers. We first grouped the brood in each nest into full sister groups using a maximum likelihood method [44] (software Kinship 1.2, available at http://www.gsoftnet.us/GSoft.html). The full-sister groups could have no more than three alleles at a given locus (if the mother was heterozygous, and differed from the father), and all females had to share the same haploid allele from their haploid father. Then we compared the genotypes of these full-sister groups to all foundresses and their mates in the population. Assignment of brood was used for two purposes. First, we determined the general reproductive structure of nests, i.e. which foundresses reproduced and how much. Second, we augmented the census data by identifying progeny on nests by foundresses that were never observed on those nests. When brood were found that could not be assigned to any of the collected foundresses, or that could not be offspring of any of the censused but uncollected foundresses, they were assumed to be brood of unknown non-resident foundresses. Finally, we also grouped the 46 foundresses sampled from our study nests to full-sister groups using Kinship 1.2.

## Supporting Information

Table S1 **Permanent moves of** ***P. carolina*** **foundresses to other nests.** Movements of wasps were observed in the field, except for wasp 45.6, which was detected from genetic data. Date is the date the focal wasp was last seen in the original nest; #W and #C are the numbers of wasps (movers included) and cells in the **original** and **target** nests, respectively, at the day of the move; #B is the number of brood foundress had laid in their original and target nests, D and S refer to the focal wasp being dominant or subordinate in their nest, wasps 41 and 42 shared dominance in the target nest 44; *R* is relatedness of the moving foundress to other foundresses in her original or target nest: *R* = FS, foundresses are full sisters, *R* = NR, foundresses are unrelated, A = foundress was alone in her original nest. Asterisk after FS or NR means that relatedness is determined by deducing from relatedness and movement patterns; no entry means that relatedness could not be determined.
(DOCX)

Table S2 **Visits of** ***P. carolina*** **foundresses in other nests.** Visitors were either observed in the target nest or genetically detected for having laid eggs there (marked with *). Date is the date the visit occurred (estimated for egg-laying visitors); #W and #C are the numbers of wasps (visitors included) in her original or target nests; *R* is relatedness of the visiting foundress to other foundresses in her original and target nests: *FS* = foundresses are full sisters, NR = foundresses are unrelated, A = foundress was alone in her permanent nest. Asterisk after FS means that relatedness is determined by deducing from relatedness and movement patterns; no entry means that relatedness could not be determined. In their original nests, wasps 9 and 19 were dominants, 12 and 20 were subordinates and 25 was alone. All wasps were subordinates in their target nests.
(DOCX)

## References

[1] Hamilton WD (1964a) The genetical evolution of social behaviour. I. J Theor Biol 7: 1–16. 5875341 10.1016/0022-5193(64)90038-4

[2] Hamilton WD (1964b) The genetical evolution of social behaviour. II. J Theor Biol 7: 17–52. 5875340 10.1016/0022-5193(64)90039-6

[3] Queller DC (1989) The evolution of eusociality: Reproductive head start of workers. Proc Natl Acad Sci U S A 86: 3224–3226. 16594034 10.1073/pnas.86.9.3224 PMC287102

[4] Queller DC (1996) The origin and maintenance of eusociality: the advantage of extended parental care. In: Turillazzi S, West-Eberhard MJ. Natural history and evolution of paper wasps. Oxford: Oxford Univeristy Press. 218–234.

[5] Queller DC, Strassmann JE (1998) Kin selection and social insects. BioScience 48: 165–175.

[6] Reeve HK (1991) *Polistes*. In: Ross KG, Matthews RW. The social biology of wasps Ithaca: Cornell University Press. 99–148.

[7] Rosengren R, Sundström L, Fortelius W (1993) Monogyny and polygyny in *Formica* ants: the result of alternative dispersal tactics. In: Keller L. Queen number and sociality in insects. New York: Oxford University press. 308–333.

[8] Pardi L (1942) Ricerche sui polistini: V. La poliginia iniziale de *Polistes gallicus* (L.). Boll Entomol Bologna 14: 104.

[9] Pardi L (1948) Dominance order in *Polistes* wasps. Physiol Zool 21: 1–13. 18898533 10.1086/physzool.21.1.30151976

[10] Seppä P, Queller DC, Strassmann JE (2002) Social conventions, competition, contracts and skew in young colonies of the social wasp, *Polistes carolina* . Behav Ecol 13: 531–542.

[11] Hughes CR, Strassmann JE (1988) Age is more important than size in determining dominance among workers in the primitively eusocial wasp, *Polistes instabilis.* . Behaviour 107: 1–14.

[12] Strassmann JE, Queller DC, Hughes CR (1987) Constraints on independent nesting by *Polistes* foundresses in Texas. In: Eder J, Remboldt H. Chemistry and biology of social insects. München: Verlag J. Peperny. 379–380.

[13] Reeve HK, Ratnieks FLW (1993) Queen-queen conflicts in polygynous societies: mutual tolerance and reproductive skew. In: Keller L. Queen number and sociality in insects. New York: Oxford University press. 45–85.

[14] Johnstone RA (2000) Models of reproductive skew: a review and synthesis. Ethology 106: 5–26.

[15] Nonacs P (2006) The rise and fall of transactional skew theory in the model genus *Polistes* . Ann Zool Fennici 43: 443–455.

[16] Strassmann JE, Hughes CR (1986) Latitudinal variation in protandry and protogyny in Polistinae wasps. Monitore Zool Ital 20: 87–100.

[17] Strassmann JE (1981) Evolutionary implication of early male and satellite nest production in *Polistes exclamas* colony cycles. Behav Ecol Sociobiol 8: 55–64.

[18] Starks PT (1998) A novel sit and wait reproductive strategy in social wasps. Proc R Soc London B 265: 1407–1410.

[19] Starks PT (2001) Alternative reproductive tactics in the paper wasp *Polistes dominulus* with specific focus on the sit-and-wait tactic. Ann Zool Fennici 38: 189–199.

[20] Gamboa GJ (1996) Kin recognition in social wasps. In: Turillazzi S, West-Eberhard MJ. Natural history and evolution of paper-wasps. Oxford: Oxford University Press. 161–177.

[21] Nonacs, P & Reeve HK (1995) The ecology of cooperation in wasps: causes and consequences of alternative reproductive decisions. Ecology 76: 953–967.

[22] Field J, Solís CR, Queller DC, Strassmann JE (1998) Social and genetic structure of paper-wasp co-foundress associations: tests of reproductive skew models, Am Nat. 151: 545–563. 10.1086/286140 18811376

[23] Lorenzi MC, Cervo R (1995) Usurpations and late associations in the solitary founding social wasp, *Polistes biglumis bimaculatus* (Hymenoptera, Vespidae). J Insect Behav 8: 443–451.

[24] Seppä P, Fogelqvist J, Gyllenstrand N, Lorenzi MC (2011) Colony kin structure in the social wasp, *Polistes biglumis*: Multiple matings by multiple foundresses. Insectes Sociaux 58: 345–355.

[25] Nonacs P, Reeve HK (1993) Opportunistic adoption of orphaned nests in paper wasps as an alternative reproductive strategy. Behav Processes 30: 47–60. 24896471 10.1016/0376-6357(93)90011-F

[26] Field J, Foster W, Shreeves G, Sumner S (1998) Ecological constraints on independent nesting in facultatively eusocial hover wasps. Proc R Soc London B 265: 973–977.

[27] Field J, Shreeves G, Sumner S (1999) Group size, queuing and helping decisions in facultatively eusocial hover wasps. Behav Ecol Sociobiol 45: 378–385.

[28] Hagen RH, Smith DR, Rissing SW (1988) Genetic relatedness among co-foundresses of two desert ants, *Veromessor pergandei* and *Acromyrmex versicolor* . Psyche 95: 191–201.

[29] Rissing SW, Pollock GB (1988) Pleometrosis and polygyny in ants. In: Jeanne RL. Interindividual Behavioural Variability in Social Insects. Boulder: Westview Press. 179–221.

[30] Hughes CR, Queller DC, Negrón-Sotomayor JA, Strassmann JE, Solis C, Gastreich KR (1993) The maintenance of high genetic relatedness in multi-queen colonies of social wasps. In: Keller L. Queen number and sociality in insects. New York: Oxford University press. 152–169.

[31] Gadagkar R, Chandrashekara K, Chandran S and Bhagavan S (1993) Serial polygyny in the primitively eusocial wasp *Ropalidia marginata*: implications for the evolution of sociality. In: Keller L. Queen number and sociality in insects. New York: Oxford University press. 188–214.

[32] Michener CD (1974) The social behavior of the bees: a comparative study. Cambridge: Harvard University Press. 404.

[33] Crozier RH, Pamilo P (1996) Evolution of Social Insect Colonies, Sex Allocation and Kin Selection. Oxford: Oxford University Press. 306 p.

[34] Sundström L, Seppä P, Pamilo P (2005) Genetic population structure and dispersal patterns in *Formica* ants – a review. Ann Zool Fennici 42: 163–177.

[35] Greenwood PJ (1980) Mating systems, philopatry and dispersal in birds and mammals. Anim Behav 28: 1140–1162.

[36] Storz JE (1999) Genetic consequences of mammalian social structure. J Mammalogy 80: 553–569.

[37] Pusey AE, Packer C (1987) The evolution of sex-biased dispersal in lions. Behaviour 101: 275–310.

[38] Gilbert DA, Packer C, Pusey AE, Stephens JC, O'Brien SJ (1991) Analytical DNA Fingerprinting in Lions: Parentage, Genetic Diversity, and Kinship. J Heredity 82: 378–386. 10.1093/oxfordjournals.jhered.a111107 1940281

[39] Heinsohn RG (2004) Parental care, load-lightening and costs. In Koenig WD, Dickinson JL. Ecology and Evolution of Cooperative Breeding in Birds. Cambridge: Cambridge University Press. 67–80.

[40] Koenig WD, Haydock J (2004) Incest and incest avoidance. In Koenig WD, Dickinson JL. Ecology and Evolution of Cooperative Breeding in Birds. Cambridge: Cambridge University Press. 142–156.

[41] Temple HJ, Hoffman JI, Amos W (2009) Group structure, mating system and extra-group paternity in the co-operatively breeding White-breasted Thrasher *Ramphocinclus brachyurus* . Ibis 151: 99–112.

[42] Strassmann JE, Barefield K, Solís CR, Hughes CR, Queller DC (1997) Trinucleotide microsatellite loci for a social wasp, *Polistes* . Mol Ecol 6: 97–100. 9004523 10.1046/j.1365-294x.1997.00158.x

[43] Strassmann JE, Solís CR, Peters JM, Queller DC (1996) Strategies for finding and using highly polymorphic DNA microsatellite loci for studies of genetic relatedness and pedigrees. In: Ferraris J, Palumbi S. Methods in Zoology and Evolution. New York: Wiley-Liss. 163–180.

[44] Goodnight KF, Queller DC (1999) Computer software for performing likelihood tests of pedigree relationships using genetic markers. Mol Ecol 8: 1231–1234. 10447863 10.1046/j.1365-294x.1999.00664.x

