# Supplementary material for: Why Wasp Foundresses Change Nests: Relatedness, Dominance, and Nest Quality
Source: PLoS One. 2012 Sep 25;7(9):e45386. doi: 10.1371/journal.pone.0045386 (PMC3458021; doi:10.1371/journal.pone.0045386)
Supplement: Table S1 — Permanent moves of P. carolina foundresses to other nests. Movements of wasps were observed in the field, except for wasp 45.6, which was detected from genetic data. Date is the date the focal wasp was last seen in the original nest; #W and #C are the numbers of wasps (movers included) and cells in the original and target nests, respectively, at the day of the move; #B is the number of brood foundress had laid in their original and target nests, D and S refer to the focal wasp being dominant or subordinate in their nest, wasps 41 and 42 shared dominance in the target nest 44; R is relatedness of the moving foundress to other foundresses in her original or target nest: R = FS, foundresses are full sisters, R = NR, foundresses are unrelated, A = foundress was alone in her original nest. Asterisk after FS or NR means that relatedness is determined by deducing from relatedness and movement patterns; no entry means that relatedness could not be determined. (DOCX) [file pone.0045386.s001.docx]

**Table S1.** Permanent moves of *P. carolina* foundresses to other nests

Movements of wasps were observed in the field, except for wasp 45.6, which was detected from genetic data. Date is the date the focal wasp was last seen in the original nest; #W and #C are the numbers of wasps (movers included) and cells in the **original** and **target** nests, respectively, at the day of the move; #B is the number of brood foundress had laid in their original and target nests, D and S refer to the focal wasp being dominant or subordinate in their nest, wasps 41 and 42 shared dominance in the target nest 44; *R* is relatedness of the moving foundress to other foundresses in her original or target nest: *R*=FS, foundresses are full sisters, *R*=NR, foundresses are unrelated, A=foundress was alone in her original nest. Asterisk after FS or NR means that relatedness is determined by deducing from relatedness and movement patterns; no entry means that relatedness could not be determined.

|  |  |  | **Original nest** | | | | | **Target nest** | | | | |
| --- | --- | --- | --- | --- | --- | --- | --- | --- | --- | --- | --- | --- |
|  | Wasp | Date | Nest | #W | #C | R | #B | Nest | #W | #C | R | #B |
| Switchers | | | | | | | | | | | | |
| 1 | 41 | March 8 | 45 | 5 | 0 | FS | 0^S^ | 44 | 1 | 0 | A | 9^D^ |
| 2 | 45.6 | March 22 | 45 | 5 | 5 | FS* | 0^S^ | 8 | 4 | 11 | NR* | 1^S^ |
| 3 | 24 | March 23 | 43 | 4 | 18 | FS* | - | 35 | 5 | 19 | FS | 0^S^ |
| 4 | 37 | March 24 | 15 | 4 | 16 | NR | 0^S^ | 39 | 8 | 34 | FS | 6^S^ |
| 5 | 45.6 | March 27 | 8 | 4 | 22 | NR* | 1^S^ | 44 | 3 | 8 | FS* | 0^S^ |
| 6 | 19.1 | April 2 | 19 | 4 | 18 | FS* | 1^S^ | 43 | 4 | 21 | NR* | - |

| **Deserters** | | | | | | | | | | | | |
| --- | --- | --- | --- | --- | --- | --- | --- | --- | --- | --- | --- | --- |
| 1 | 42 | March 24 | 7 | 1 | 8 | A | - | 44 | 2 | 5 | FS | 9^D^ |
| 2 | 41.2 | March 27 | 41 | 1 | 7 | A | - | 35 | 6 | 28 | FS* | 0^S^ |
| 3 | 2.2 | April 2 | 2 | 1 | 11 | A | - | 3 | 2 | 10 |  | - |
| 4 | 119.2 | April 12 | 119 | 1 | 12 | A | - | 19 | 4 | 21 | FS* | - |
| 5 | 39 | April 13 | 43 | 1 | 36 | A | - | 42 | 1 | 18 | A | 3^D^ |
| 6 | 25 | April 21 | 32 | 1 | 19 | A | - | 35 | 8 | 42 | FS | 1^S^ |
