# Supplementary material for: Why Wasp Foundresses Change Nests: Relatedness, Dominance, and Nest Quality
Source: PLoS One. 2012 Sep 25;7(9):e45386. doi: 10.1371/journal.pone.0045386 (PMC3458021; doi:10.1371/journal.pone.0045386)
Supplement: Table S2 — Visits of P. carolina foundresses in other nests. Visitors were either observed in the target nest or genetically detected for having laid eggs there (marked with *). Date is the date the visit occurred (estimated for egg-laying visitors); #W and #C are the numbers of wasps (visitors included) in her original or target nests; R is relatedness of the visiting foundress to other foundresses in her original and target nests: FS = foundresses are full sisters, NR = foundresses are unrelated, A = foundress was alone in her permanent nest. Asterisk after FS means that relatedness is determined by deducing from relatedness and movement patterns; no entry means that relatedness could not be determined. In their original nests, wasps 9 and 19 were dominants, 12 and 20 were subordinates and 25 was alone. All wasps were subordinates in their target nests. (DOCX) [file pone.0045386.s002.docx]

**Table S2.** Visits of *P. carolina* foundresses in other nests.

Visitors were either observed in the target nest or genetically detected for having laid eggs there (marked with *). Date is the date the visit occurred (estimated for egg-laying visitors); #W and #C are the numbers of wasps (visitors included) in her original or target nests; *R* is relatedness of the visiting foundress to other foundresses in her original and target nests: *FS*=foundresses are full sisters, NR=foundresses are unrelated, A=foundress was alone in her permanent nest. Asterisk after FS means that relatedness is determined by deducing from relatedness and movement patterns; no entry means that relatedness could not be determined. In their original nests, wasps 9 and 19 were dominants, 12 and 20 were subordinates and 25 was alone. All wasps were subordinates in their target nests.

|  |  |  | **Original Nest** | | | | **Target Nest** | | | |
| --- | --- | --- | --- | --- | --- | --- | --- | --- | --- | --- |
|  | Wasp id | Date | Nest id | #W | #C | R | Nest id | #W | #C | R |
| 1 |  | March 23 |  |  |  |  | 32 | 2 | 14 |  |
| 2* | 19d | March 26 |  |  |  |  | 19 | 5 | 8 | FS* |
| 3* | 12 | March 26 | 26 | 2 | 12 | FS | 23 | 3 | 8 | NR |
| 4* | 26a | March 26 |  |  |  |  | 26 | 3 | 12 | FS* |
| 5* | 20 | March 26 | 33 | 3 | 16 | FS | 29 | 4 | 12 | FS |
| 6* | 29a | March 26 |  |  |  |  | 29 | 4 | 12 | FS* |
| 7* | 31a | March 26 |  |  |  |  | 31 | 5 | 19 | FS* |
| 8* | 19 | March 26 | 33 | 3 | 16 | FS | 31 | 5 | 19 | NR |
| 9* | 36b | March 26 |  |  |  |  | 36 | 3 | 11 | FS* |
| 10* | 25 | March 26 | 32 | 1 | 16 | A | 43 | 4 | 24 | FS* |
| 11* | 44a | March 26 |  |  |  |  | 44 | 2 | 7 | FS* |
| 12 | 42.6 | April 11 |  |  |  |  | 42 | 2 | 18 |  |
| 13 |  | April 11 |  |  |  |  | 43 | 3 | 36 |  |
| 14 | 12 | April 12 | 26 | 2 | 17 | FS | 23 | 3 | 12 | NR |
| 15 |  | April 13 |  |  |  |  | 36 | 3 | 12 |  |
| 16 | 9 | April 14 | 15 | 3 | 18 | FS | 14 | 6 | 30 | NR |
| 17 |  | April 14 |  |  |  |  | 33 | 4 | 23 |  |
| 18 |  | April 14 |  |  |  |  | 44 | 4 | 18 |  |
| 19 |  | April 14 |  |  |  |  | 45 | 3 | 23 |  |
| 20* | 20 | April 14 | 33 | 3 | 23 | FS | 29 | 3 | 16 | FS |
| 21 | 15.5 | April 21 |  |  |  |  | 15 | 3 | 18 |  |
| 22 |  | April 21 |  |  |  |  | 39 | 9 | 46 |  |
